# Paternal phthalate exposure-elicited offspring metabolic disorders are associated with altered sperm small RNAs in mice

**DOI:** 10.1016/j.envint.2023.107769

**Published:** 2023-01-23

**Authors:** Jingwei Liu, Junchao Shi, Rebecca Hernandez, Xiuchun Li, Pranav Konchadi, Yuma Miyake, Qi Chen, Tong Zhou, Changcheng Zhou

**Affiliations:** a Division of Biomedical Sciences, School of Medicine, University of California, Riverside, CA 92521, United States; b Department of Physiology and Cell Biology, University of Nevada, Reno School of Medicine, NV 89557, United States

**Keywords:** Phthalate, Paternal exposure, Transgenerational inheritance, Metabolic disease, Sperm small non-coding RNA, PANDORA-seq

## Abstract

Exposure to ubiquitous plastic-associated endocrine disrupting chemicals (EDCs) is associated with the increased risk of many chronic diseases. For example, phthalate exposure is associated with cardiometabolic mortality in humans, with societal costs ~ $39 billion/year or more. We recently demonstrated that several widely used plastic-associated EDCs increase cardiometabolic disease in appropriate mouse models. In addition to affecting adult health, parental exposure to EDCs has also been shown to cause metabolic disorders, including obesity and diabetes, in the offspring. While most studies have focused on the impact of maternal EDC exposure on the offspring’s health, little is known about the effects of paternal EDC exposure. In the current study, we investigated the adverse impact of paternal exposure to a ubiquitous but understudied phthalate, dicyclohexyl phthalate (DCHP) on the metabolic health of F1 and F2 offspring in mice. Paternal DCHP exposure led to exacerbated insulin resistance and impaired insulin signaling in F1 offspring without affecting diet-induced obesity. We previously showed that sperm small non-coding RNAs including tRNA-derived small RNAs (tsRNAs) and rRNA-derived small RNAs (rsRNAs) contribute to the intergenerational transmission of paternally acquired metabolic disorders. Using a novel PANDORA-seq, we revealed that DCHP exposure can lead to sperm tsRNA/rsRNA landscape changes that were undetected by traditional RNA-seq, which may contribute to DCHP-elicited adverse effects. Lastly, we found that paternal DCHP can also cause sex-specific transgenerational adverse effects in F2 offspring and elicited glucose intolerance in female F2 descendants. Our results suggest that exposure to endocrine disrupting phthalates may have intergenerational and transgenerational adverse effects on the metabolic health of their offspring. These findings increase our understanding of the etiology of chronic human diseases originating from chemical-elicited intergenerational and transgenerational effects.

## 1. Introduction

Numerous endocrine disrupting chemicals (EDCs) that are used in plastic production have become a significant health concern (Eriksen et al., 2014; Seltenrich, 2015). Plastic base chemical bisphenol A (BPA), and many phthalate plasticizers, are associated with increased metabolic disease risk in humans (Helsley and Zhou, 2017; Lang et al., 2008; Lind and Lind, 2012; Lind and Lind, 2011; Melzer et al., 2012; Melzer et al., 2010; Olsen et al., 2012; Trasande et al., 2022; Vom Saal and Myers, 2008). The adverse health effects of BPA have been extensively studied, and phthalate exposure has also been shown to be associated with increased cardiometabolic disease risk and mortality in humans, with societal costs ~$39 billion/year or more (Casals-Casas and Desvergne, 2011; Halden, 2010; Lind et al., 2012; Trasande et al., 2022; Wu et al., 2021). However, the mechanisms by which exposure to those endocrine disrupting phthalates influences cardiometabolic disease are not well understood.

In addition to affecting adult health, parental exposure to a wide range of environmental toxicants including plastic-associated EDCs can cause metabolic disorders, including obesity and diabetes, and these metabolic disease risks can be transmitted to their offspring (Heindel and Blumberg, 2019; Lee and Blumberg, 2019; Sales et al., 2017). Studies from worm to mammal suggest environmental stress-induced phenotypes can be “memorized” in the germline and transmitted to future generations (Daxinger and Whitelaw, 2012; Heard and Martienssen, 2014; Lane et al., 2014; Ost et al., 2014). Most data are obtained from the impact of maternal exposure on the offspring health. For example, we previously provided the first evidence that maternal murine exposure to BPA exacerbates atherosclerosis in their adult offspring (Sui et al., 2018). However, emerging evidence suggests epigenetic inheritance can also occur between father and offspring. A subsequent wave of research searches for sperm regulatory factors that could relay this transmission of phenotype in a non-DNA sequence-based manner (Chen et al., 2016). We and others have recently demonstrated that sperm small non-coding RNAs (sncRNAs) including rRNA-derived small RNAs (rsRNAs) and tRNA-derived small RNAs (tsRNAs) form the ‘sperm RNA code’ to carry important epigenetic information that can induce intergenerational transmission of paternally acquired metabolic disorders (Chen et al., 2016; Sarker et al., 2019; Zhang et al., 2019; Zhang et al., 2018).

Compared to other well-studied sncRNAs (e.g., microRNAs or miR-NAs), many tsRNAs and rsRNAs have RNA modifications from their precursors and may show different RNA termini due to cleavage by different RNase (Phizicky and Hopper, 2010; Schimmel, 2018; Sergiev et al., 2018; Shi et al., 2021; Shi et al., 2022). When performing RNA-seq analysis for highly modified sperm sncRNAs, certain RNA modifications (including RNA methylations and terminal RNA modifications) can interfere with the cDNA library construction process (Cozen et al., 2015; Honda et al., 2015; Zheng et al., 2015), which prevents discovery of highly modified tsRNAs and rsRNAs that may be responsible for transmitting paternal phenotypes (Shi et al., 2022). To address this problem, we have successfully developed an innovative RNA-seq protocol, PANDORA-seq (Panoramic RNA Display by Overcoming RNA Modification Aborted Sequencing) to overcome RNA modification-elicited sequence interferences (Shi et al., 2021).

In the current study, we investigated the effects of paternal exposure to a ubiquitous phthalate, dicyclohexyl phthalate (DCHP) on the metabolic health of F1 and F2 offspring in mice. We also performed PANDORA-seq to reveal the paternal DCHP exposure-elicited sperm tsRNA and rsRNA changes.

## 2. Materials and methods

### 2.1. Animals

**Fig. 1.**
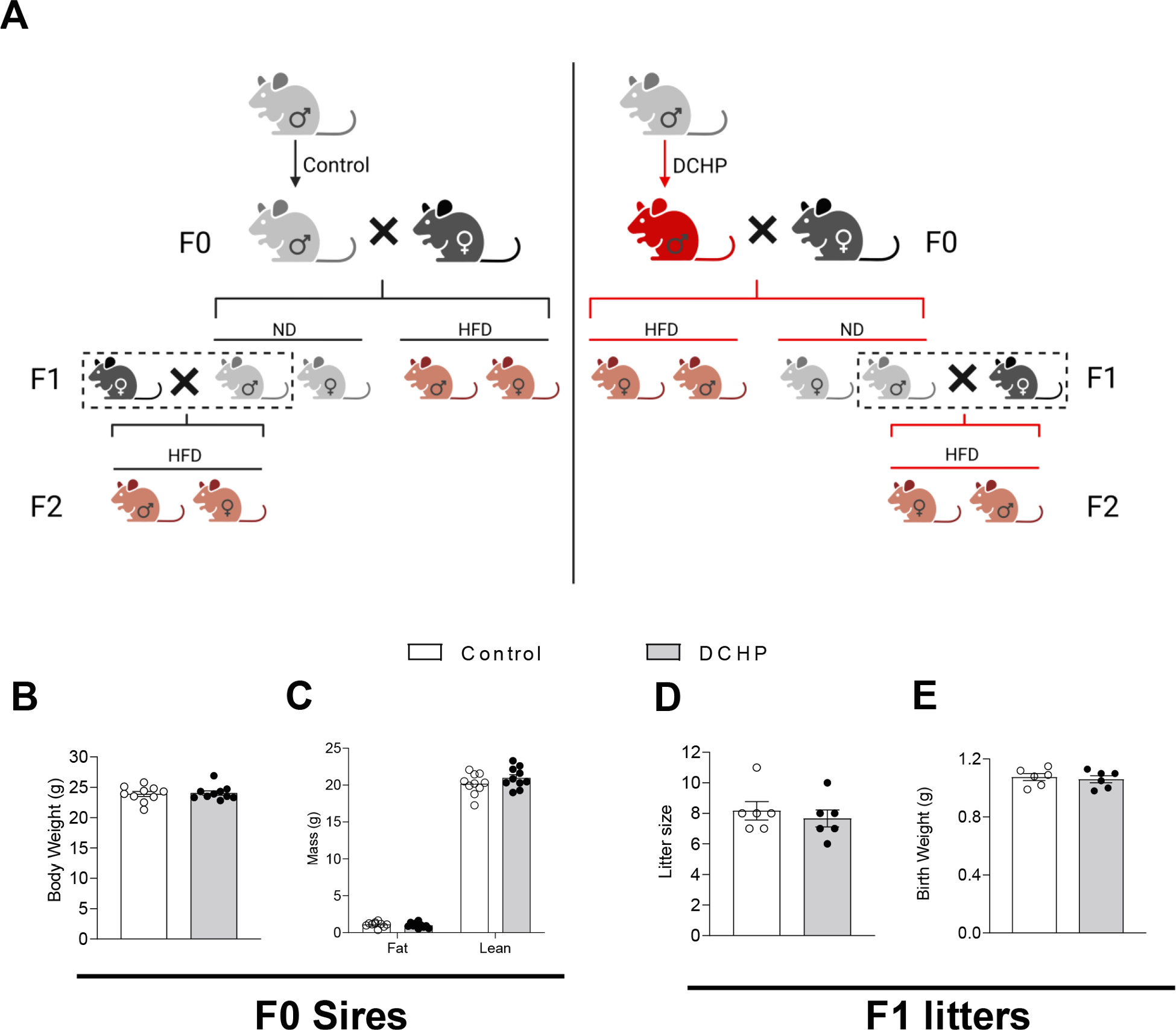
Scheme of paternal DCHP exposure study and the impact of DCHP exposure on F0 sires and F1 litters. (A) Eight-week-old male C57BL/6 wild-type (WT) mice were treated with vehicle control or 10 mg/kg/day of DCHP by oral gavage for 4 weeks. Control or DCHP-exposed male mice were mated with 12-week-old unexposed female WT mice. The F1 offspring were weaned at 3-week-old and were fed a normal chow diet (ND) or a high-fat diet (HFD) for 9 weeks. ND-fed male F1 mice then mated with 12-week-old unexposed female WT mice to generate F2 offspring that were also fed HFD for 9 weeks after weaning. (B and C) The impact of DCHP exposure on body weight (B) and lean and fat mass (C) of F0 sires (n = 9–10, two-sample, two tailed Student’s *t*-test). (D and E) The impact of paternal DCHP exposure on the litter size (D) and average birth weight (E) of F1 offspring (n = 6, two-sample, two tailed Student’s *t*-test).

Eight-week-old male wild-type (WT) mice (C57BL/6J strain, purchased from the Jackson Laboratory) were fed a normal chow diet (ND; PicoLab Rodent Diet 20, Lab Supply) containing 20 % protein and 4.5 % fat by weight (Calories: 25 % protein, 13 % Fat, 62 % Carbohydrate) and received daily oral gavage of corn oil vehicle control or DCHP (10 mg/kg body weight, Sigma-Aldrich) for 4 weeks. At 12 weeks old, the mice were mated with 12-week-old unexposed female WT mice (C57BL/6J strain, purchased from the Jackson Laboratory). After copulation was confirmed by vaginal plug detection, the male mice were removed from the mating cage and humanely euthanized. These initial mouse pairs were designated F0. The F0 dams were kept on the ND. The F1 offspring were weaned at 3 weeks and provided with either a ND or Western-type high-fat diet (HFD, TD.88137, Envigo) containing 17.3 % protein and 21 % fat by weight (Calories: 15 % protein, 42 % fat, and 42 % carbohydrate) (Helsley et al., 2016; Lu et al., 2019) until euthanasia at 12 weeks old. Before euthanasia, the F1 male mice on ND were mated with 12-week-old unexposed WT females (C57BL/6J strain, purchased from the Jackson Laboratory) to generate F2 generation. The F2 mice were also weaned at 3 weeks old and fed with the same HFD for 9 weeks before euthanasia (Fig. 1A). Mice were restricted to food for 6 h prior to euthanasia, and tissue collection was performed as described in previous publication (Sui et al., 2021; Wang et al., 2018). All animal studies followed protocols approved by the University of California, Riverside Institutional Animal Care and Use Committee.

### 2.2. Metabolic phenotypic analyses

Body weight was recorded weekly. NMR spectroscopy (EchoMRI) was performed before euthanasia to measure the lean and fat mass of the mice (Helsley et al., 2016; Lu et al., 2019). We performed glucose tolerance test (GTT) and insulin tolerance test (ITT) as described in previous studies (Helsley et al., 2016; Sui et al., 2014). For insulin stimulation studies, mice were injected with 0.35 U/kg body weight Humulin R U-100 insulin (Lilly USA. LLC) into the inferior vena cava (Helsley et al., 2016). Mice were euthanized after 5 min and major tissues (skeletal muscle, liver, white adipose fat) were collected for further analysis (Helsley et al., 2016).

### 2.3. Sperm isolation

We collected mouse sperm from the F0 sires at the end of treatment using a previously described procedure (Shi et al., 2021). After release from the cauda epididymis, sperm were incubated for 15 min (37 °C) in 1 × phosphate-buffered saline (PBS). Tissue debris was removed by filtering the sperm through a cell strainer (pore size 40-μm, Corning, CLS431750). Somatic cell residues were eliminated by incubating the sperm with somatic cell lysis buffer for 40 min on ice. The precipitation of sperm was achieved through centrifugation (600 × g, 5 min). Afterwards, sperm was washed twice with 1 × PBS solution and precipitated again at 600 × g, 5 min. The sperm was finally resuspended in 1 mL TRIzol Reagent (Thermo Fisher Scientific 15596026) and stored at −80 °C until further use.

### 2.4. Western blotting

We used previously described methods for the extraction of protein and immunoblotting analyses (Helsley et al., 2016; Sui et al., 2021). The denatured protein lysates were resolved using SDS-PAGE. The separated proteins were transferred onto a nitrocellulose membrane (Bio-Rad, 1620115). After blocking with 5 % bovine serum albumin (BSA, Sigma-Aldrich, A9647) for 1 h at room temperature, the membranes were incubated with primary antibodies overnight at 4 °C: anti-Actin (1:5000 dilution, Millipore Sigma A2066), anti-Akt (1:1000 dilution, Cell Signaling 9272), and anti-Phospho-Akt (Ser473) antibodies (1:1000 dilution, Cell Signaling 9271). After 4 × 5 min washes with 1 × PBS with 0.1 % Tween20 (PBST), the membranes were incubated for 1 h with an anti-rabbit secondary antibodies (1:5000 dilution, Millipore Sigma 12–348) at room temperature. After 4 × 5 min washes, the membranes were developed by Pierce ECL Western Blotting Substrate kit (Thermo Fisher Scientific, 32209), and exposed with Bio-Rad Chemidoc imaging machine. Uncropped immunobloting images are included in Supplemental Figure 1.

### 2.5. RNA extraction and quantitative Real-Time PCR assay

TRIzol Reagent (Thermo Fisher Scientific 15596026) was used to perform total RNA extraction from mouse tissues following manufacturer instructions (Satta et al., 2022; Shi et al., 2021; Sui et al., 2021). We measured the relative mRNA expression levels by Quantitative Real-Time PCR with the SYBR Green (Bio-Rad 170–8886) kit using a Bio-Rad CFX Real-Time-PCR Machine (184–5096) (Satta et al., 2022; Sui et al., 2021). The primer sequences are included in Supplemental Table 1.

### 2.6. RNA-seq and transcriptomic data analysis

After isolation RNA integrity was checked using a Bioanalyzer (Agilent Technologies Inc., Santa Clara, CA). The Illumina standard operation pipeline was used for cDNA library construction and sequencing as described previously (Meng et al., 2022; Shi et al., 2021; Sui et al., 2020), as were the detailed RNA-seq data analysis methods (Meng et al., 2022; Shi et al., 2021; Sui et al., 2020). The cut-off threshold for differential expression was set as fold change (FC) > 1.5 and a false discovery rate (FDR) < 0.1. Further, Gene Ontology (GO) Biological process analysis proceeded with the differentially expressed genes as previously described (Meng et al., 2022; Sui et al., 2020). The raw transcriptome datasets have been uploaded and can be accessed in the Gene Expression Omnibus (GSE215807).

### 2.7. PANDORA-seq of sperm small RNAs

We recently developed the PANDORA-seq protocol (Shi et al., 2021). Detailed information for PANDORA-seq of sperm small RNAs is also described as following.

Small RNA extraction and cDNA library creation were done using our recently published protocol (Shi et al., 2021). Briefly, RNA loading dye from New England Biolabs (B0363S) was added to the sperm RNA sample, mixed well, and incubated for 5 min at 75 °C. The RNA mixture was electrophoresed through a urea polyacrylamide gel (15 %). SYBR Gold solution (Invitrogen; S11494) was used to stain and visualize the small RNA. The excised small RNA (15–50 nucleotides) was eluted overnight with sodium acetate (0.3 M, Invitrogen, AM9740) at 4 °C. After 10 min centrifugation (12,000 × g, 4 °C), the aqueous phase was collected. Subsequently, linear acrylamide (Invitrogen, AM9520), sodium acetate (3 M) and ethanol (100 %) were added into the collected supernatant and incubated for 2 h at −20 °C. The RNA was precipitated and resuspended in Nuclease-free H_2_O.

200 ng sperm RNA was added into 50 μL reaction buffer, which consists of α-ketoglutaric acid (1 mM, Sigma–Aldrich; K1128), AlkB (4 μg mL^−1^), sodium ascorbate (2 mM), HEPES (50 mM, Fisher Scientific, 15630080), BSA (50 mg L^−1^, Sigma-Aldrich, A9647), ferrous ammonium sulfate (75 μM), and RNase inhibitor (2,000 U mL^−1^). The mixture was incubated for 20 min (37 °C), after which 500 μL TRIzol reagent was added into the mixture to extract the RNA. The isolated RNA was transferred into another reaction buffer (50 μL) containing 10 × PNK buffer (5 μL, New England Biolabs, B0201S), ATP (1 mM, New England Biolabs, P0756S), T4PNK (10 U, New England Biolabs, M0201L) and incubated for 20 min (37 °C). 500 μL TRIzol was added for RNA extraction.

NEBNext Small RNA Library Prep Set for Illumina kit (New England Biolabs; E7330S) was used to conduct small RNA library construction. Detailed PCR parameters were published previously (Shi et al., 2021). PAGE gel was run for purification of the PCR product. Qualified libraries were amplified and sent for sequencing at the Genomics Center of University of California, San Diego (Illumina system, SE75 strategy). The software *SPORTS1.1* (parameter setting: -M 1) was used to analyze and annotate the raw sequencing outputs (Shi et al., 2018; Shi et al., 2021). To investigate the relative abundance of sncRNAs, we normalized the expression of the individual sncRNA species by total miRNA expression (Shi et al., 2021). The 20,000 sncRNA species with the highest normalized mean expression were retained. The samr package was then applied to identify the differentially expressed sncRNAs (based on the normalized expression) between DCHP-exposed and control groups. The sncRNA species with *q*-value < 10 % and FC > 2 were deemed differentially expressed. The sperm RNA sequence datasets have been uploaded and can be accessed in the Gene Expression Omnibus (GSE215807).

### 2.8. Statistical analysis

All data are displayed in the format of the mean ± standard error (SE). Two-sample, two-tailed Student’s *t*-test was used for individual pairwise comparisons. Two-way ANOVA was applied when multiple comparisons were performed (with Bonferroni correction method). Data analyses were performed using GraphPad Prism software with the statistically significant level set at *p* < 0.05.

## 3. Results

### 3.1. Paternal exposure to phthalate DCHP had no effect on litter size or birth weight of F1 offspring

To investigate the potential effects of paternal DCHP exposure on offspring health, 8-week-old male WT mice were fed a normal chow diet (ND) and treated with vehicle control or 10 mg/kg body weight/day of DCHP by oral gavage for 4 weeks before mating with 12-week-old unexposed female WT mice (Fig. 1A). After the vaginal plug was detected (embryonic day 0.5), pregnant females were housed separately and fed the ND. The sires were euthanized for tissue and sperm collection. Exposure to DCHP did not affect the body weight of sires (Fig. 1B). We also measured their body composition by Echo MRI (NMR spectroscopy) and found that DCHP exposure affected neither lean nor fat mass of these mice (Fig. 1C). In addition, paternal DCHP treatment affected neither litter size nor birth weight of F1 offspring (Fig. 1D and 1E).

### 3.2. Paternal DCHP exposure leads to exacerbated insulin resistance and impaired insulin signaling in F1 offspring without affecting diet-induced obesity

**Fig. 2.**
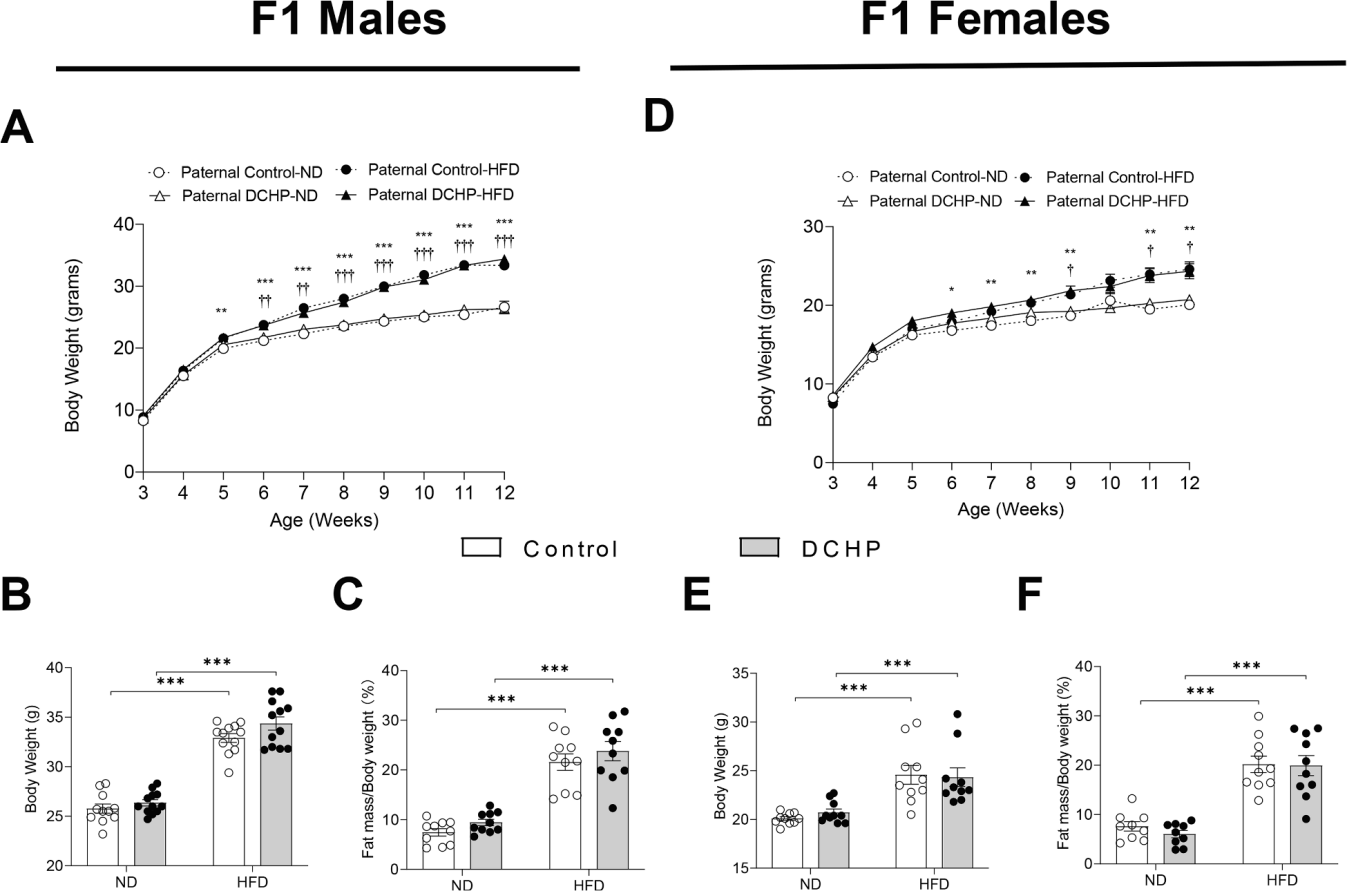
Paternal DCHP exposure does not affect diet-induced obesity in the F1 offspring. Eight-week-old male WT mice were treated with vehicle control or 10 mg/kg/day of DCHP by oral gavage for 4 weeks. F1 descendants of control or DCHP-exposed sires were fed a ND or HFD for 9 weeks. Growth curve (A and D), final body weight (B and E), and fat mass (percentage of body weight) (C and F) of male and female F1 mice were measured (n = 9–12, two-way *ANOVA* followed by Bonferroni’s multiple comparison test). Statistically significant differences between ND and HFD fed F1 mice from control sires were indicated with * (*P < 0.05, **P < 0.01, and ***P < 0.001). Statistically significant differences between ND and HFD fed F1 mice from DCHP sires were indicated with † (†*P* < 0.05, ††*P* < 0.01, and †††*P* < 0.001).

F1 pups were weaned on postnatal day 21 and fed a ND or high-fat diet (HFD) (Helsley et al., 2016; Lu et al., 2019; Sui et al., 2014; Wang et al., 2018) for 9 weeks before euthanasia at 12 weeks of age. To determine whether paternal DCHP exposure affected diet-induced obesity in the offspring, the body weight of male and female F1 offspring was measured weekly. In addition, body composition was analyzed by EchoMRI. Body weight and fat mass were significantly increased following HFD feeding in both male and female F1 mice. However, paternal DCHP exposure had no effect on the growth curve or body composition (e.g., lean and fat mass) of ND or HFD-fed male and female F1 offspring (Fig. 2). Therefore, paternal DCHP exposure did not affect diet-induced obesity in the F1 offspring.

**Fig. 3.**
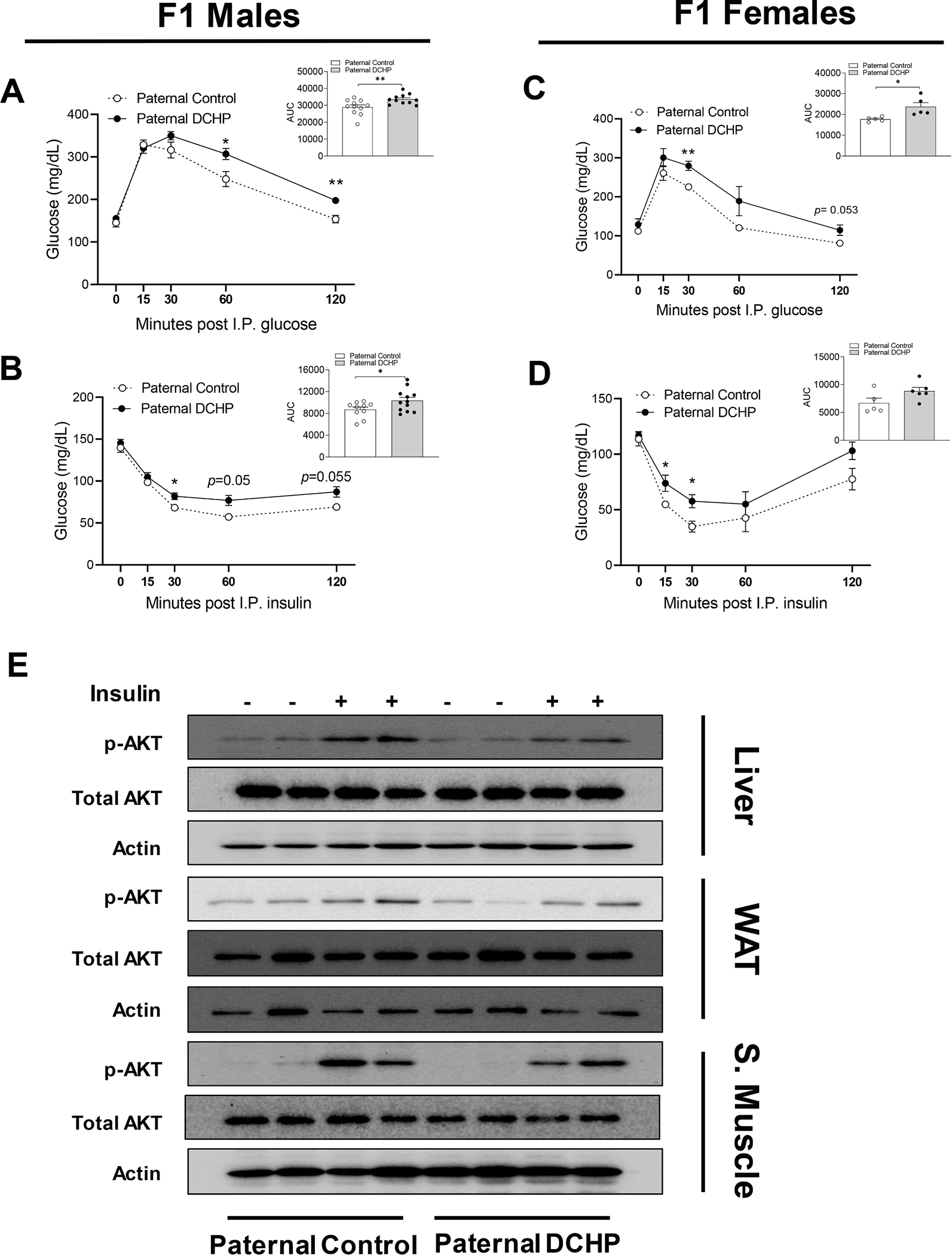
Paternal DCHP exposure leads to exacerbated diabetic phenotypes and impaired insulin signaling in HFD-fed F1 offspring. Eight-week-old male WT mice were treated with vehicle control or 10 mg/kg/day of DCHP by oral gavage for 4 weeks. F1 descendants of control or DCHP-exposed sires were fed a HFD for 9 weeks. (A-D) Glucose tolerance test (GTT) and the area under the curve (AUC) of GTT (A and C), and insulin tolerance test (ITT) and AUC of ITT (B and D) in HFD-fed F1 male and female offspring (n = 5–12, two-sample, two tailed Student’s *t*-test, *P < 0.05 and **P < 0.01). (E) Immunoblotting of phosphorylated Akt and total Akt levels in the liver, white adipose tissue (WAT), and skeletal muscle of F1 male mice injected with saline or 0.35 units/kg body weight insulin (n = 3).

Despite similar body weight and adiposity, paternal DCHP exposure led to increased diabetic phenotypes in F1 offspring. We performed glucose and insulin tolerance tests (GTT & ITT) in those mice and found that both male and female offspring from paternal DCHP-exposed sires had worse glucose tolerance (Fig. 3A and 3C) and exhibited reduced hypoglycemic response to administered insulin (Fig. 3B and 3D). To understand the impact of paternal DCHP exposure on insulin signaling in obese F1 offspring, HFD-fed F1 male mice were treated with insulin prior to euthanasia, and phosphorylated Akt levels were evaluated in multiple tissues. We found that paternal DCHP exposure impaired Akt phosphorylation in response to insulin in liver, skeletal muscle, and white adipose tissue (WAT) (Fig. 3E), which indicates impaired insulin signaling in multiple tissues of the offspring.

### 3.3. Paternal exposure to DCHP induces hepatic transcriptomic changes in F1 offspring

**Fig. 4.**
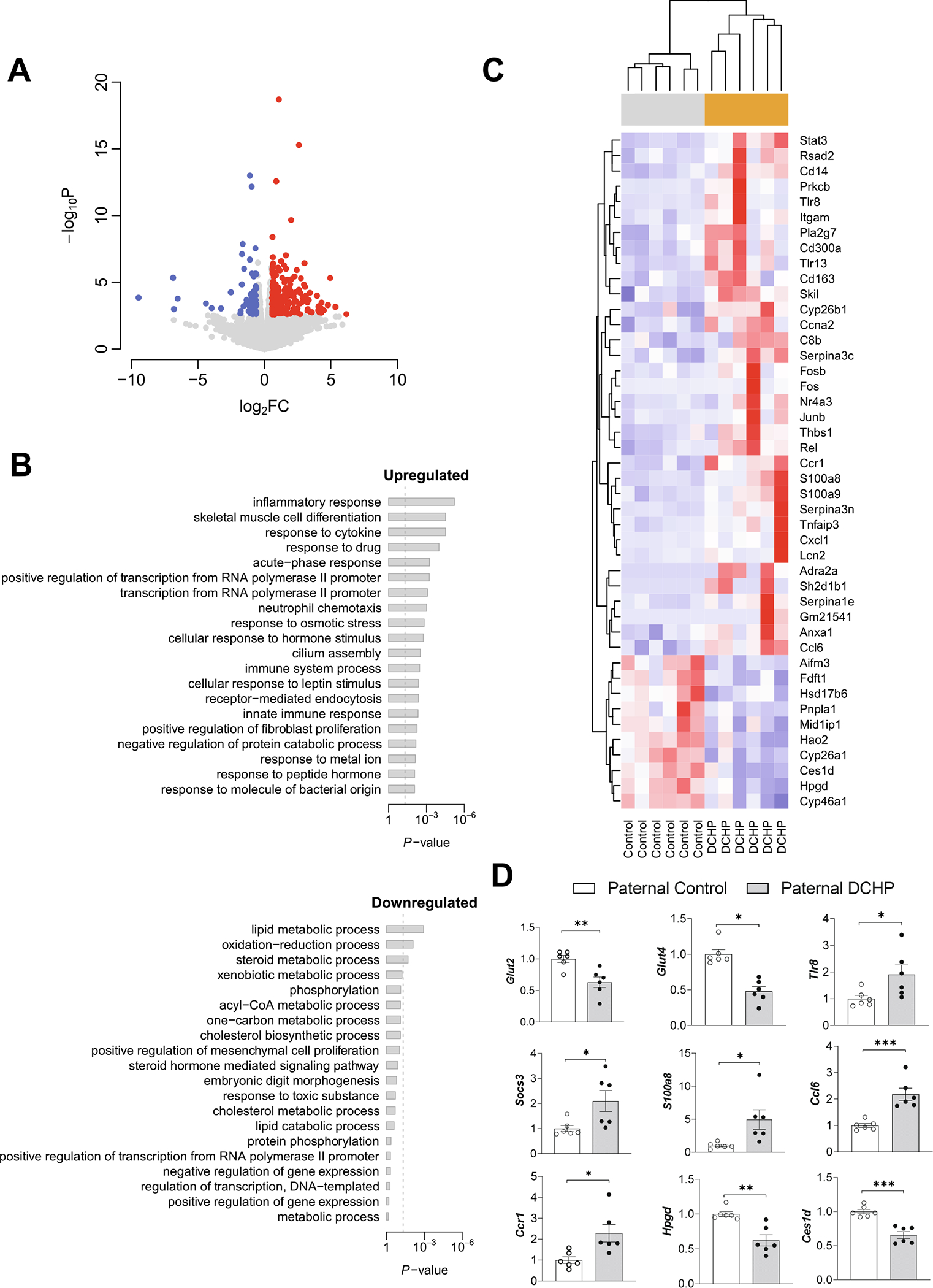
Paternal DCHP exposure alters hepatic transcriptome in F1 male offspring. Eight-week-old male WT mice were treated with vehicle control or 10 mg/kg/day of DCHP by oral gavage for 4 weeks. F1 descendants of control or DCHP-exposed sires were fed a HFD for 9 weeks. Total RNAs were extracted from the liver of F1 male mice for RNA-seq analysis. (A) Volcano plot of differentially expressed genes (DEGs) between F1 descendants of DCHP-exposed sires and F1 descendants of control sires. Colored dots represent the up-regulated (red) and down-regulated (blue) hepatic DEGs of F1 offspring with a false discovery rate (FDR) < 0.1 and a fold change (FC) > 1.5 as a cut-off threshold. (B) Gene Ontology (GO) Biological Process terms associated with the hepatic DEGs of F1 offspring. The *P*-values were computed by *Fisher*’s exact test. The vertical dash line indicates the significance level of *α* = 0.05. The y-axis displays the GO terms while the x-axis displays the adjusted *P*-values. (C) Heatmap representation of DEGs involved in the GO Biological Processes of “inflammatory response”, “lipid metabolism process”, “cellular response to leptin stimulus”, “immune system process”, “oxidation–reduction process’, “neutrophil chemotaxis”, “response to cytokine’, and “ cellular response to hormone stimulus” shown in panel B. Each row shows one individual genes and each column shows a biological replicate of mouse. Red represents relatively increased gene expression while blue denotes downregulation. (D) QPCR analyses of representative hepatic genes of F1 offspring (n = 6, two-sample, two tailed Student’s *t*-test, *P < 0.05, **P < 0.01, and ***P < 0.01). (For interpretation of the references to colour in this figure legend, the reader is referred to the web version of this article.)

To further understand transcriptomic changes in key organs (e.g., liver) from paternal DCHP exposure that may have contributed to the metabolic disorders of F1 offspring, we isolated total RNA from the liver of F1 males and performed RNA sequencing (RNA-seq) analysis. Liver plays a central role in whole-body glucose homeostasis (Lin and Accili, 2011; Nordlie et al., 1999) and previous studies also reported that paternal EDC exposure can affect key hepatic signaling in the offspring to increase insulin resistance (Gong et al., 2021). RNA-seq results showed that paternal DCHP exposure led to 289 differentially expressed genes (DEGs, 227 upregulated and 62 downregulated) in the liver of F1 offspring with a cut-off threshold of fold change (FC) > 1.5 and false discovery rate (FDR) < 0.1 (Fig. 4A and Supplemental Table 2). Gene Oncology (GO) Biological Process analysis was then performed and the results revealed DEGs were enriched in biological processes related to metabolic disease risk including “inflammatory response”, “lipid metabolism process”, and “cellular response to leptin stimulus”. (Fig. 4B and C) (Hernandez and Zhou, 2021; Hotamisligil and Erbay, 2008; Saengnipanthkul et al., 2021). The hepatic DEGs associated with these pathways were either upregulated or downregulated in F1 offspring of DCHP-exposed sires (Fig. 4C and Supplemental Table 2).

We next performed QPCR analysis to verify the expression changes of several DEGs discovered by RNA-seq analysis. Paternal DCHP exposure consistently caused significantly increased hepatic expression of genes known to be associated with inflammation or metabolic disorders (e.g., *Tlr8*, *Ccl6*, *Ccr1*, and *S100a8*) (Fig. 4D) (Ahmad et al., 2016; Catalán et al., 2011; Jiao et al., 2009; Lylloff et al., 2017; Zeyda and Stulnig, 2009). By contrast, several genes known to promote insulin sensitivity (e.g., *Hpgd* and *Ces1d*) (Fig. 4D) (Li et al., 2022; Quiroga et al., 2012; Schmidleithner et al., 2019) were downregulated in the liver of F1 offspring of DCHP-exposed sires. Further, QPCR analyses showed paternal DCHP exposure led to decreased hepatic expression of *Glut2* and *Glut4*, two key genes involved in hepatic glucose homeostasis (Fig. 4D). Collectively, these results suggest paternal DCHP exposure leads to altered hepatic genes and pathways associated with insulin resistance or metabolic disorders.

### 3.4. PANDORA-seq unveils paternal DCHP exposure-elicited sperm tsRNA and rsRNA changes

We and others have demonstrated that sperm sncRNAs, including tsRNAs and rsRNAs, can sensitively respond to environmental exposures and act as causative agents in mediating offspring’s metabolic phenotypes (Chen et al., 2016; Frye et al., 2018; Zhang et al., 2016; Zhang et al., 2019; Zhang et al., 2018). However, many of these sncRNAs bear various RNA modifications, preventing the discovery of highly modified sperm tsRNAs and rsRNAs in widely used traditional RNA-seq methods (Shi et al., 2021; Shi et al., 2022). To overcome this obstacle, we developed a novel small RNA sequencing method, PANDORA-seq, to eliminate RNA modification-elicited sequence interferences (Shi et al., 2021).

**Fig. 5.**
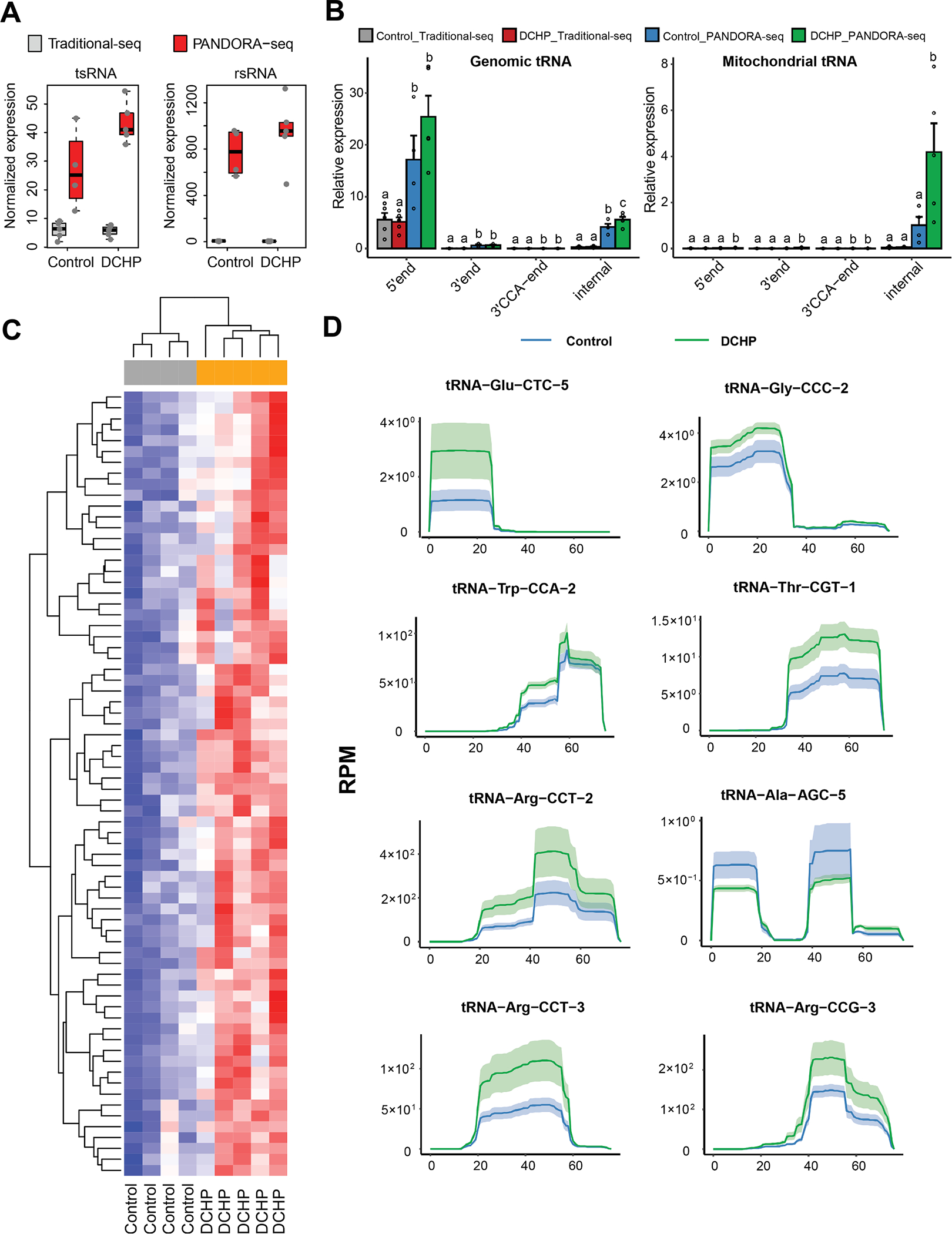
PANDORA-seq identifies significantly changed tsRNAs and rsRNAs in the sperm of DCHP treated sires. Eight-week-old male WT mice were treated with vehicle control or 10 mg/kg/day of DCHP by oral gavage for 4 weeks. Total RNAs were isolated from the sperms of F0 male mice and used for PANDORA-seq or traditional small RNA sequencing. (A) Normalized tsRNA and rsRNA (normalized to miRNAs) under traditional-seq and PANDORA-seq protocols. (B) tsRNA responses to traditional-seq and PANDORA-seq corresponding to different origins (5′ tsRNA, 3′ tsRNA, 3′ tsRNA-CCA end, and internal tsRNAs). The y axes represent the relative expression levels compared with total reads of miRNAs. Statistical significance was determined by two-sided one-way ANOVA with uncorrected Fisher’s LSD test. Bars bearing different letters above were significantly different from each other (*P* < 0.05). All data are plotted as means ± SEM (n = 4–5 in each group). (C) Relative expression heatmap of the differentially expressed tsRNAs and rsRNAs in the sperm of control or DCHP-exposed mice detected by PANDORA-seq (n = 4–5). Each row shows one individual tsRNA or rsRNA and each column shows a biological replicate of mouse. Red represents higher relative expression while blue denotes lower relative expression. (D) Dynamic responses to vehicle control or DCHP of representative individual tsRNAs detected by PANDORA-seq. The solid curves indicate the mean reads per million (RPM) values for the control and DCHP-exposed groups. The colored bands represent the 95 % confidence interval (n = 4–5). (For interpretation of the references to colour in this figure legend, the reader is referred to the web version of this article.)

To determine whether PANDORA-seq can detect novel sperm sncRNAs that may confer metabolic dysfunction in the offspring of DCHP-exposed sires, total RNAs isolated from sperms of control and DCHP-exposed sires were subjected to both traditional RNA-seq and PANDORA-seq, and the sequencing data were analyzed by *SPORTS1.1* bioinformatics analysis software (Shi et al., 2018; Shi et al., 2021). Consistent with our previous studies (Shi et al., 2021), PANDORA-seq revealed an overall rsRNA- and tsRNA-enriched sperm sncRNA landscape that was not detected by traditional RNA-seq (Fig. 5A). The origins of sperm tsRNAs in regards to their loci from tRNA precursors including 3′tsRNAs, 5′tsRNAs, 3′tsRNAs with a CCA end, and internal tsRNAs, were also analyzed. PANDORA-seq revealed an increased relative expression (normalized to miRNAs) of specific tsRNA origins as compared to traditional RNA-seq (Fig. 5B). Further, exposure to DCHP induced differentially expressed tsRNAs and rsRNAs was detected only by PANDORA-seq but not by traditional RNA-seq, and the significantly changed tsRNA and rsRNA sequences are shown in the heatmap (Fig. 5C and Supplemental Table 3). Consistently, mapping of tsRNA expression patterns on individual tRNA length scales (e.g., tRNA-Glu-CTC, tRNA-Arg-CCT) revealed that the tsRNAs also contain distinct dynamic responses to DCHP treatment (Fig. 5D). These results showed that PANDORA-seq can detect more sperm tsRNAs and rsRNAs that were otherwise undetectable using the traditional RNA-seq method, consistent with previous results (Shi et al., 2021). The functions of individual tsRNAs or rsRNAs are mostly unknown, but their overall changed signature after DCHP treatment may have functional consequences that contribute to DCHP-induced intergenerational metabolic disorders.

### 3.5. Paternal exposure to DCHP leads to impaired glucose tolerance in F2 female mice

**Fig. 6.**
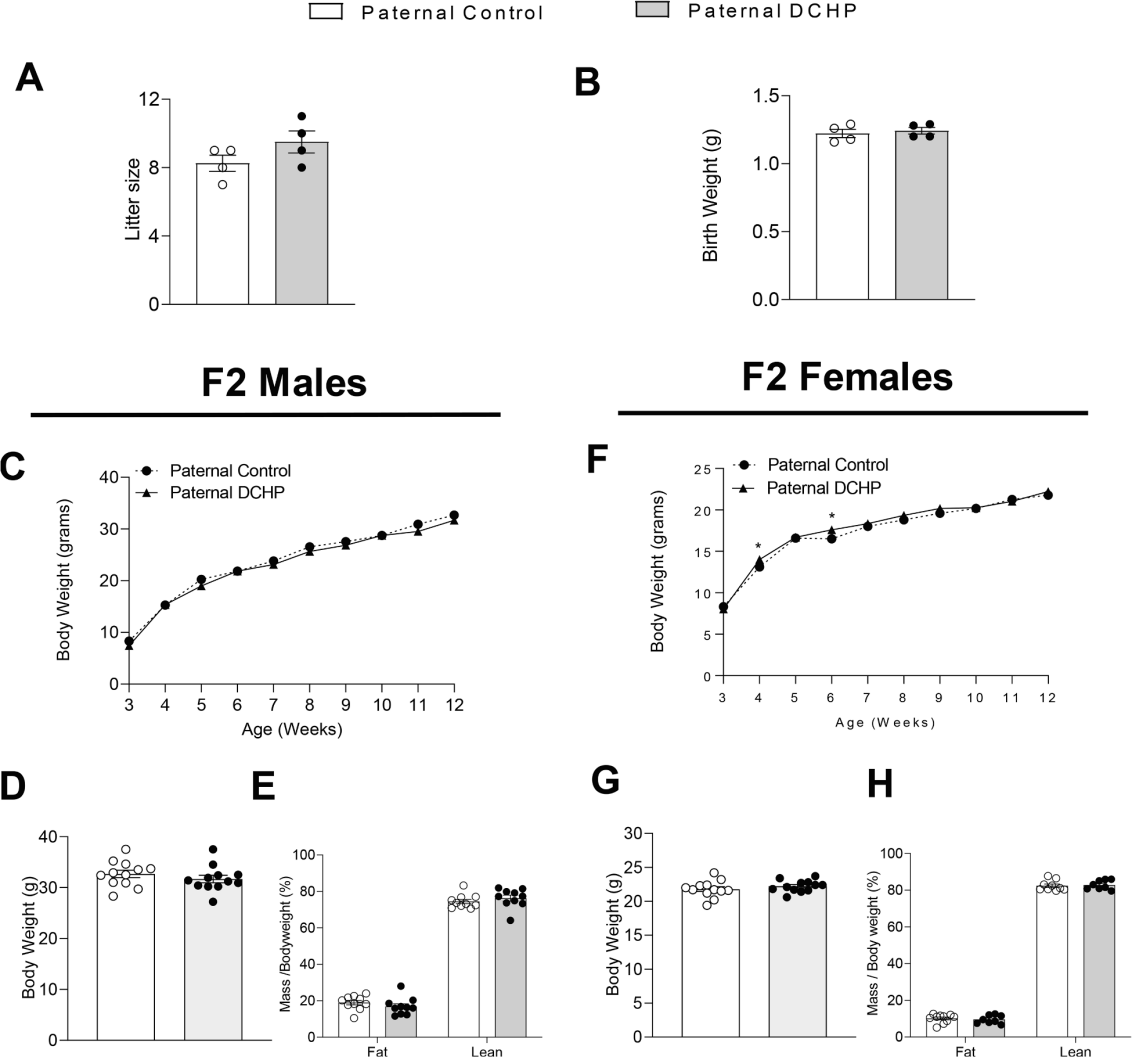
Paternal DCHP exposure does not affect the litter size, birth weight, and growth curves of F2 offspring. F2 descendants of control or DCHP-exposed sires were fed a HFD for 9 weeks. Litter size (A) and average birth weights (B) of the F2 mice (n = 4). The growth curves, final body weight, and lean and fat mass (percentage of body weight) of the F2 male (C-E) and female (F-H) offspring (n = 8–12, two-sample, two tailed Student’s *t*-test, *P < 0.05).

In addition to F1 offspring, we also mated ND-fed 12-week-old F1 males to age-matched unexposed female WT mice to generate F2 offspring. After weaning, F2 litters were fed the same HFD as the F1 descendants for 9 weeks. Similar to F1 offspring, paternal DCHP exposure had no effect on litter size or birth weight of F1 offspring (Fig. 6A and B). In addition, paternal DCHP exposure affected neither diet-induced obesity nor body composition, including lean and fat mass, in HFD-fed male and female F2 offspring (Fig. 6A–H).

**Fig. 7.**
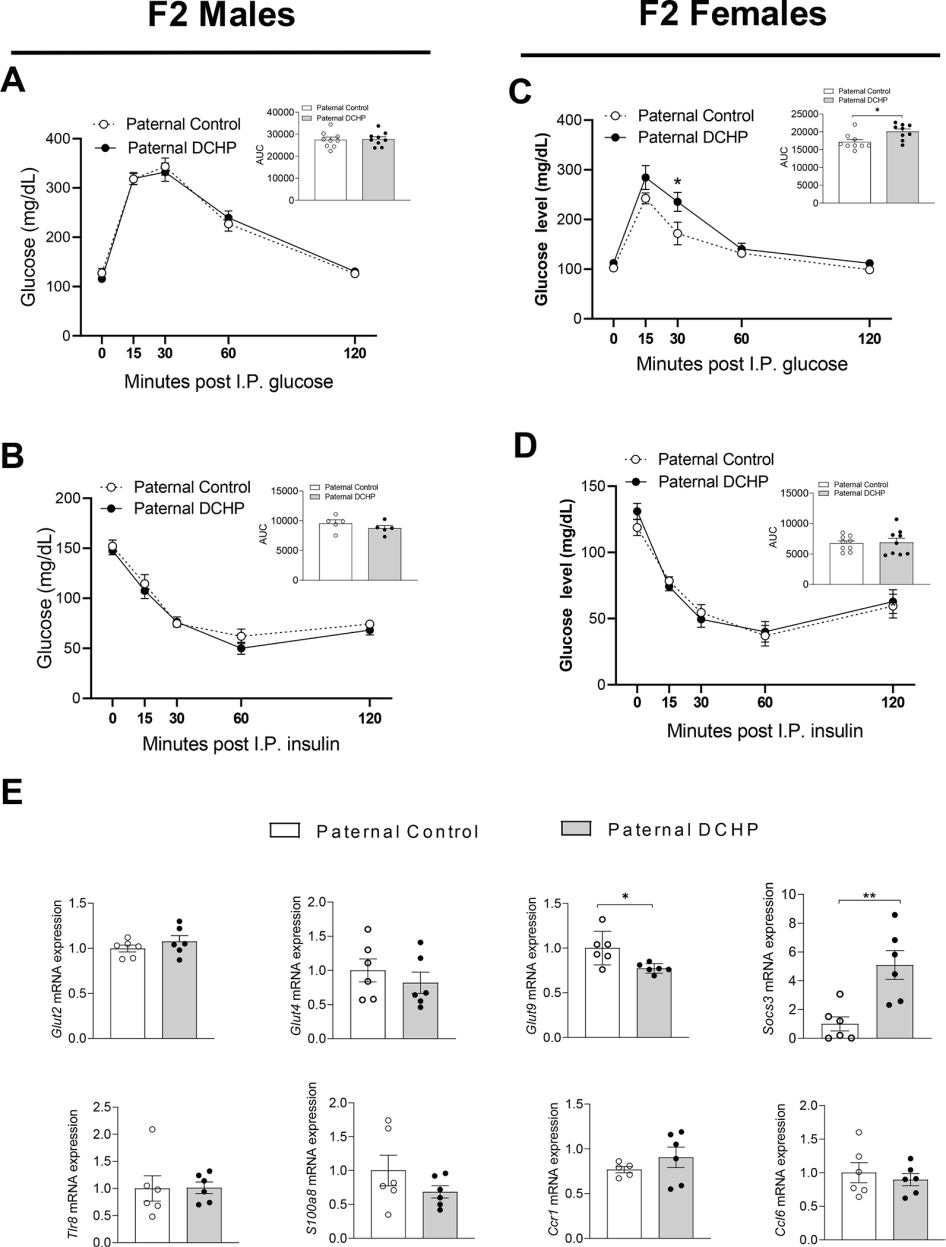
Paternal DCHP exposure elicits sex-specific transgenerational effects in F2 offspring. F2 descendants of control or DCHP-exposed sires were fed a HFD for 9 weeks. (A-D) Glucose tolerance test (GTT) and the area under the curve (AUC) of GTT (A,C), and insulin tolerance test (ITT) and AUC of ITT (B,D) in HFD-fed F2 offspring (n = 5–9, two-sample, two tailed Student’s *t*-test, *P < 0.05). (E) QPCR analyses of hepatic gene expression of F2 female mice (n = 6, two-sample, two tailed Student’s *t*-test, *P < 0.05 and ** P < 0.01).

Interestingly, female but not male F2 mice from DCHP exposed F0 sires had impaired glucose tolerance, but comparable insulin tolerance results, as compared to F2 mice from unexposed sires (Fig. 7A–D), suggesting paternal DCHP exposure has sex-specific transgenerational effects on offspring metabolic health. We analyzed hepatic gene expression to understand the potential mechanisms underlying the glucose intolerant phenotype in F2 female mice. Several genes, including *Glut 2* and *Tlr8*, that were altered by paternal DCHP exposure in F1 mice were unchanged in F2 mice (Fig. 7E). However, we found that another glucose transporter family gene, *Glut9*, was significantly downregulated by paternal DCHP exposure in the liver of F2 female mice (Fig. 7E). Further, paternal DCHP exposure significantly increased the hepatic expression of *Socs3* in F2 females, consistent with the expression patten in F1 offspring (Fig. 7E). These results suggest that paternal DCHP exposure can have transgenerational impact on hepatic gene expression that may contribute to the observed metabolic disorders.

## 4. Discussion

Mounting evidence demonstrates exposure to EDCs can cause numerous adverse health effects in humans (Casals-Casas and Desvergne, 2011; Colborn et al., 1996; Diamanti-Kandarakis et al., 2009; Gore et al., 2015; Gross, 2007; Heindel et al., 2017; Helsley and Zhou, 2017; Kavlock et al., 1996). One challenge in risk assessment for EDC exposure is that individuals may or may not display health issues from the direct EDC exposure, but the adverse effects of these ancestral exposures may manifest in their non-exposed offspring. In our current study, we investigated the adverse effects of paternal exposure to DCHP, a widely used phthalate, on offspring metabolic health. We found that paternal DCHP exposure can induce intergenerational metabolic disorders in the F1 offspring. Using an innovative PANDORA-seq method, we revealed that DCHP exposure can lead to sperm tsRNA and rsRNA landscape changes undetected by traditional RNA-seq. Paternal DCHP exposure-elicited metabolic phenotype in the F1 offspring can be transmitted to F2 offspring, as F2 female descendants from DCHP-exposed sires also developed glucose intolerance. To the best of our knowledge, the current study is the first one demonstrating that paternal exposure to endocrine disrupting phthalates can induce intergenerational and transgenerational metabolic disorders in the offspring.

Previous studies have demonstrated the detrimental effects of parental exposure to plastic-associated EDCs, including BPA and several phthalates, on the long-term health outcome of progeny (Bansal et al., 2017; Brehm and Flaws, 2019; Li et al., 2014; Meltzer et al., 2015; Rattan et al., 2018; Zhou et al., 2017). These adverse effects include altered anogenital distance (Mammadov et al., 2018; Miao et al., 2011; Sun et al., 2018), impaired glucose/insulin tolerance (Li et al., 2014; Mao et al., 2015), altered neonatal behavior (Engel et al., 2008), and cardiometabolic disorders (Liu et al., 2022). However, most evidence has been obtained from maternal exposure studies, and little is known about the impact of paternal EDC exposure on offspring health. Further, BPA and several other phthalates (e.g., DEHP) have been extensively studied, and their adverse health effects have attracted considerable attention (Casals-Casas and Desvergne, 2011; Clara et al., 2010; Fierens et al., 2012; Halden, 2010). However, the impact of exposure to DCHP on human health are poorly understood. DCHP is used in numerous products and can also be detected in food, water, and indoor particulate matter (Cao et al., 2015; Cheng et al., 2016; EPA, 2019; Sakhi et al., 2014; Schecter et al., 2013). DCHP and its metabolites can also be found in human urinary and blood samples, and DCHP exposure levels can be high in certain populations (Blount et al., 2000; Hartmann et al., 2018; Huang et al., 2014; Saravanabhavan et al., 2013; Wang et al., 2013). Therefore, EPA has recently designated DCHP as one of twenty high-priority substances for risk evaluation (EPA, 2019). Our study revealed the intergenerational and transgenerational effects of paternal DCHP exposure on the F1 and F2 offspring. Therefore, future studies should be conducted on the adverse health effects of these understudied phthalates.

While paternal DCHP exposure induced metabolic disorders in both male and female F1 offspring, it is intriguing that female but not male F2 offspring from DCHP-exposed sires showed impaired glucose tolerance phenotype. These results indicated that paternal DCHP exposure can lead to sex-specific transgenerational effects on the metabolic health of their progenies. Previous human epidemiological studies and animal studies have reported sexual dimorphic responses to early life perturbations, from either ancestral side (Aiken and Ozanne, 2013; Sandovici et al., 2022; Vallaster et al., 2017; Vickers et al., 2011). Most of the existing paradigms regarding sex-specific inheritance traits are intergenerational, whereas evidence regarding transgenerational sex-different transmission is very limited. For example, Gong et al. found that paternal inorganic arsenic exposure elicited hepatic insulin resistance and glucose intolerance in F1 female but not male mice (Gong et al., 2021). Similarly, another study showed only female offspring of HFD-fed mice displayed glucose intolerance and resistance to diet-induced obesity (de Castro Barbosa et al., 2016). By contrast, Maloney et al., reported that maternal methyl-deficient diet feeding during the *peri*-conception period led to impaired glucose homeostasis only in male offspring (Maloney et al., 2011). Another study reported that paternal chronic social defeat preferentially affects the locomotor activity and sucrose preference in male offspring but the altered depression- and anxiety-related behavior was observed in both genders (Dietz et al., 2011). Our current study only used F1 males to breed with unexposed female mice to generate F2 offspring. It would be interesting to investigate whether F2 offspring generated by mating F1 females with unexposed male mice have similar sex-specific metabolic phenotypes in the future. While the underlying mechanisms for sex-specific inheritance are largely unknown, some hypotheses have been proposed, including epigenetic modifications on sex chromosomes (Pembrey et al., 2006), sex differences related to mitochondiral DNA and sncRNAs (Godschalk et al., 2017; Wang et al., 2022a; Wang et al., 2022b), and sex hormone-induced differential gene expression (Carone et al., 2010; Tramunt et al., 2020; Waxman and O’Connor, 2006). Therefore, future studies are required to test those hypotheses and understand how parental exposure affects sex-specific effects on the offspring’s health.

To understand how paternal DCHP exposure affects genes or pathways in key organs that may contribute to the observed phenotypes in the offspring, we performed RNA-seq and QPCR analyses of hepatic genes in F1 mice. Our results revealed the altered expression of key genes or pathways by paternal DCHP exposure. In addition to reduced glucose transporters (e.g., *Glut2* and *Glut4*), we also found higher expression of inflammatory genes or inflammatory pathway genescores in the liver of F1 mice from paternal DCHP-exposed sires. It has been well-established that chronic inflammation contributes to the pathogenesis of metabolic disorders or insulin resistance (Gregor and Hotamisligil, 2011; Hernandez and Zhou, 2021; Kahn et al., 2006; Shoelson et al., 2006). Similar to our findings, Bansal *et al.*, previously reported that maternal exposure to BPA impaired insulin secretion and increased proinflammatory cytokines in F1 and F2 male mice (Bansal et al., 2017). Paternal DCHP exposure led to increased expression of hepatic *Socs3* in both F1 and F2 offspring. Socs3 can be induced by certain inflammatory cytokines (e.g., TNFα and IL6) to contribute to inflammation-mediated insulin resistance in the liver and adipose tissue (Cao et al., 2018; Jorgensen et al., 2013; Torisu et al., 2007). It is plausible that *Socs3* and other inflammatory genes contribute to paternal DCHP exposure-elicited metabolic phenotypes in the offspring.

While accumulating evidence shows that paternal environmental input (e.g*.,* dietary manipulation, exercise, toxicant exposure) may confer lifetime metabolic phenotype alterations in offspring (Gong et al., 2021; Oluwayiose et al., 2021; Ost et al., 2014; Stanford et al., 2018; Wang et al., 2022a; Watkins et al., 2018; Zhang et al., 2021), the underlying mechanisms for paternally acquired phenotypes remain elusive. We and others have recently demonstrated that paternally acquired phenotypic traits are achieved at least partially through sperm sncRNAs (Chen et al., 2016; Gapp et al., 2014; Grandjean et al., 2015; Klastrup et al., 2019; Ord et al., 2020; Rodgers et al., 2015; Sarker et al., 2019; Wang et al., 2021; Zhang et al., 2021; Zhang et al., 2018). For example, we previously discovered that mature mouse sperms carry a dominant form of small RNAs including tsRNAs and rsRNAs (Chen et al., 2016; Peng et al., 2012; Zhang et al., 2018). We demonstrated that the injection of sperm tsRNA/rsRNA-enriched RNA fractions (30–40nt) from HFD-exposed male mice into control zygotes can lead to the development of metabolic disorders in F1 mice (Chen et al., 2016; Zhang et al., 2018). We further showed that RNA modifications can alter secondary sncRNA structures and biological properties and are required for sperm sncRNAs’ biological impact in epigenetic inheritance (Zhang et al., 2018). These studies support the concept of ‘sperm RNA code’ in programming offspring metabolic phenotypes (Chen, 2022; Zhang et al., 2019). However, many RNA modifications prevent the detection of important sncRNAs in widely used RNA-seq methods (Shi et al., 2022). To overcome this obstacle, we developed a novel RNA sequencing method, PANDORA-seq (Shi et al., 2021). In the current study, DCHP exposure led to changes in sperm tsRNAs and rsRNAs that were detectable by PANDORA-seq but not traditional RNA sequencing.

It remains unclear how DCHP causes sperm tsRNA and rsRNA changes. We recently demonstrated that DCHP is a ligand of a nuclear hormonal receptor, pregnane X receptor (PXR) and DCHP may induce adverse effects in adult mice through PXR signaling (Sui et al., 2021). It is plausible that DCHP-mediated activation of PXR or other signaling pathways may regulate biogenesis of sperm tsRNAs and rsRNA. In addition, it is also possible that DCHP altered sperm sncRNAs through indirect mechanisms. For example, previous studies demonstrated that exposure to various environmental factors including stress, malnutrition, and chemicals can alter the sperm RNA profiles (Chen et al., 2016; Gapp et al., 2014; Rodgers et al., 2013; Rodgers et al., 2015; Rompala et al., 2018). These paternal preconceptual life experiences can also lead to the dysregulation of hypothalamic–pituitaryadrenal (HPA) axis and altered physical activities in the offspring (Rodgers et al., 2013; Short et al., 2016; Short et al., 2017), which may contribute to the development of metabolic disorders (Broadney et al., 2018; Stull, 2016; Wareham et al., 2000). It would be interesting to investigate whether DCHP exposure can also affect physiological and behavioral phenotypes of the sires and their offspring in the future by characterizing HPA stress axis responsivity, prepulse inhibition, performance on the tail suspension test, or performance in the light–dark box (Rodgers et al., 2013). In addition, detailed measurements of food and water consumption, physical activities, and indirect calorimetry using metabolic cages as we previous described (Park et al., 2016; Sui et al., 2014) would help us to understand the impact of DCHP exposure on energy balance and physical activities of the sires and their offspring. DCHP-elicited unfavorable changes of those factors may lead to altered sperm sncRNAs including tsRNAs and rsRNAs. Lastly, the functions of the altered tsRNAs and rsRNAs by DCHP are also unknown. Further studies, including the isolation and zygotic injection of individual tsRNAs/rsRNAs, are needed to explore the functions of these tsRNAs/rsRNAs in mediating DCHP and other EDC-induced intergenerational and transgenerational metabolic disorders.

In summary, paternal exposure to DCHP, an EPA-designated high-priority substance for risk evaluation, can cause intergenerational and transgenerational adverse metabolic effects in F1 and F2 offspring without affecting diet-induced obesity. Paternal DCHP exposure led to impaired insulin signaling and altered hepatic gene expression in their offspring. Using a novel PANDORA-seq, we revealed that DCHP exposure can lead to sperm tsRNA/rsRNA landscape changes undetected by traditional RNA-seq, which may contribute to DCHP-elicited adverse effects. Our study suggests that exposure to ubiquitous phthalates may not only have adverse effects on adults but also increase the risk of metabolic diseases in their progeny. These findings are intended to increase our understanding of the etiology of chronic human diseases originating from chemically-elicited intergenerational effects.

## Supplementary Material

Supplementary Material

## Data Availability

Data will be made available on request.
