# Supplementary material for: Paternal phthalate exposure-elicited offspring metabolic disorders are associated with altered sperm small RNAs in mice
Source: Environ Int. Author manuscript; Available in PMC 2023 May 18. (PMC10194346; doi:10.1016/j.envint.2023.107769)

Supplementary Material for

Paternal phthalate exposure-elicited offspring metabolic disorders are associated with altered sperm small RNAs in mice

Jingwei Liu,[1] Junchao Shi,[1] Rebecca Hernandez,[1] Xiuchun Li,[1] Pranav Konchadi,[1] Yuma Miyake,[1] Qi Chen,[1] Tong Zhou,[2] and Changcheng Zhou [1,*]

[1]Division of Biomedical Sciences, School of Medicine, University of California, Riverside, CA 92521
[2]Department of Physiology and Cell Biology, University of Nevada, Reno School of Medicine, NV 89557

This PDF file includes:

Supplemental Table 1-3
Supplemental Figure 1. Uncropped Western blot images.

Supplemental Tale 1. Primer sequences for QPCR

**Genes**	**Forward Primer sequences (5'-3')**	**Reverse primer sequences(5'-3')**
glut2	GGCTAATTTCAGGACTGGTT	TTTCTTTGCCCTGACTTCCT
glut4	CAGAAGGTGATTGAACAGAG	AATGATGCCAATGAGAAAGG
glut9	CCAATATGGGTACAACGTAGCTG	GCGTCAAGGTGAAGGACTCAATA
tlr8	GAAAACATGCCCCCTCAGTCA	CGTCACAAGGATAGCTTCTGGAA
ccl6	GCTGGCCTCATACAAGAAATGG	GCTTAGGCACCTCTGAACTCTC
ccr1	CTCATGCAGCATAGGAGGCTT	ACATGGCATCACCAAAAATCCA
s100a8	AAATCACCATGCCCTCTACAAG	CCCACTTTTATCACCATCGCAA
socs3	ATGGTCACCCACAGCAAGTTT	TCCAGTAGAATCCGCTCTCCT
hpgd	GTGAACGGCAAAGTGGCTCT	TCCAATCCACCAATGCTACCT
hsd17b6	GGAGCGTGTTGGAGACAGAG	GAGGTTCACTTGAAAGATAGGCA
ces1d	AGAGCCCTGGAGCTTCGTG	GAGCACATAGGCGGGTAGGAG
cyp46a1	AGCCGCTATGAGCACATCC	CCATACTTCTTAGCCCAATCCAG
galnt2	GAGGAGGCCCTTTTCATCGC	ACCTTCAAAGCTCTCTGGTAAGA
gapdh	AACTTTGGCATTGTGGAAGG	GGATGCAGGGATGATGTTCT

Supplemental Table 2. Detailed RNA-seq data of hepatic DEGs induced by paternal DCHP exposure

Gene	logFC	PValue	FDR	FC	Gene	logFC	PValue	FDR	FC
Gm21541	6.1584	0.0025	0.0982	71.4248	***Selenbp2***	2.4367	0.0000	0.0010	5.4141
Pla2g4b	5.3363	0.0007	0.0488	40.4009	***Srrm4***	2.3779	0.0001	0.0117	5.1980
Klk1	4.9436	0.0000	0.0024	30.7727	***Lcn2***	2.3586	0.0002	0.0266	5.1287
Ngp	4.7673	0.0005	0.0406	27.2329	***Gulp1***	2.2790	0.0018	0.0812	4.8533
Fam205a1	4.4598	0.0013	0.0673	22.0052	***Ccr1***	2.2730	0.0003	0.0276	4.8333
Grip2	4.2877	0.0003	0.0309	19.5306	***Btg2***	2.2610	0.0000	0.0020	4.7933
Gm49369	4.2749	0.0019	0.0848	19.3589	***H2bc9***	2.1984	0.0009	0.0556	4.5896
Lrrc69	4.2265	0.0008	0.0544	18.7203	***Thsd7a***	2.1952	0.0001	0.0172	4.5795
Moxd1	4.1776	0.0009	0.0552	18.0960	***Mmp7***	2.1660	0.0001	0.0167	4.4879
Sh2d1b1	4.1291	0.0018	0.0812	17.4980	***Hcar2***	2.1628	0.0004	0.0332	4.4777
Shisal1	4.1172	0.0006	0.0454	17.3539	***Dhrs9***	2.1619	0.0001	0.0127	4.4750
Adra2a	4.0640	0.0007	0.0488	16.7256	***Prokr1***	2.1378	0.0017	0.0801	4.4010
Dmrta2	4.0557	0.0007	0.0485	16.6295	***Ly6d***	2.1182	0.0016	0.0755	4.3415
Actl9	4.0478	0.0013	0.0682	16.5395	***Dusp8***	2.0451	0.0000	0.0027	4.1270
Gipc2	3.9476	0.0001	0.0162	15.4297	***Chrm3***	2.0060	0.0000	0.0000	4.0167
Capn9	3.5745	0.0018	0.0812	11.9130	***Serpina5***	1.9527	0.0000	0.0102	3.8710
Myom3	3.4259	0.0001	0.0123	10.7476	***Atf3***	1.9384	0.0000	0.0052	3.8329
Egr2	3.4209	0.0016	0.0783	10.7100	***Mcf2l***	1.9204	0.0023	0.0956	3.7852
Cd247	3.3421	0.0002	0.0248	10.1407	***Tmem156***	1.8908	0.0001	0.0126	3.7085
Mmp8	3.2537	0.0010	0.0613	9.5378	***Serpina1e***	1.8735	0.0001	0.0160	3.6643
Sult1e1	3.2363	0.0014	0.0692	9.4239	***Fam222a***	1.8664	0.0000	0.0070	3.6463
Saa2	3.2276	0.0003	0.0309	9.3670	***S100a9***	1.8654	0.0003	0.0306	3.6437
Fos	3.2078	0.0002	0.0260	9.2393	***Scn3a***	1.8256	0.0004	0.0353	3.5446
Gm20422	3.1865	0.0002	0.0235	9.1041	***Syn1***	1.7870	0.0004	0.0362	3.4510
Egr1	3.1185	0.0001	0.0160	8.6846	***Mup8***	1.7404	0.0005	0.0383	3.3414
Cxcl1	3.0568	0.0000	0.0064	8.3210	***Prkcb***	1.7199	0.0009	0.0566	3.2942
Tchh	3.0085	0.0000	0.0005	8.0472	***Nr4a3***	1.6975	0.0025	0.0987	3.2435
S100a8	2.9737	0.0000	0.0052	7.8556	***Slc37a1***	1.6974	0.0000	0.0030	3.2431
Saa1	2.9241	0.0002	0.0197	7.5897	***9930111J21Rik1***	1.6970	0.0022	0.0934	3.2422
Ckap2	2.8844	0.0001	0.0184	7.3838	***E030018B13Rik***	1.6444	0.0003	0.0296	3.1261
Tff3	2.8463	0.0003	0.0296	7.1915	***Tlr8***	1.6286	0.0001	0.0190	3.0922
Ugt2b37	2.7411	0.0000	0.0025	6.6857	***1700017B05Rik***	1.6186	0.0000	0.0009	3.0708
Btbd19	2.6922	0.0004	0.0345	6.4628	***Mt2***	1.6173	0.0000	0.0073	3.0680
44807	2.6846	0.0025	0.0979	6.4288	***Abcb1b***	1.6159	0.0000	0.0002	3.0651
Epb41l3	2.6716	0.0001	0.0120	6.3713	***Fmn2***	1.5882	0.0000	0.0102	3.0067
Scara5	2.5991	0.0000	0.0000	6.0591	***Ccdc120***	1.5620	0.0010	0.0589	2.9527
Adcy1	2.5949	0.0000	0.0086	6.0415	***Vmn2r124***	1.5559	0.0020	0.0884	2.9402
Fosb	2.5905	0.0004	0.0345	6.0229	***Bdkrb2***	1.5541	0.0014	0.0702	2.9365
F13a1	2.5133	0.0019	0.0848	5.7092	***Socs3***	1.5425	0.0001	0.0136	2.9129
Myc	2.4852	0.0009	0.0555	5.5992	***Fst***	1.5335	0.0020	0.0871	2.8949

Gene	logFC	PValue	FDR	FC	Gene	logFC	PValue	FDR	FC
Igfbp1	1.5218	0.0013	0.0675	2.8716	***Fgl1***	1.0213	0.0000	0.0069	2.0297
Steap4	1.5050	0.0000	0.0096	2.8382	***Atp11a***	1.0016	0.0019	0.0833	2.0022
Aspm	1.5002	0.0000	0.0044	2.8289	***Serpina3n***	0.9912	0.0003	0.0309	1.9879
Pkib	1.4820	0.0002	0.0242	2.7934	***Rasgef1b***	0.9841	0.0001	0.0177	1.9780
Cd14	1.4766	0.0000	0.0006	2.7828	***Slc8a1***	0.9681	0.0008	0.0539	1.9562
Galnt3	1.4385	0.0020	0.0872	2.7104	***Acmsd***	0.9638	0.0017	0.0804	1.9504
Bmper	1.4061	0.0017	0.0788	2.6502	***Cyp26b1***	0.9626	0.0006	0.0424	1.9488
E2f8	1.3474	0.0000	0.0024	2.5446	***Rsad2***	0.9227	0.0002	0.0242	1.8957
Thbs1	1.3466	0.0008	0.0518	2.5431	***Fgfr1***	0.9131	0.0004	0.0347	1.8831
Tnfaip3	1.3187	0.0003	0.0309	2.4945	***C9orf72***	0.9064	0.0003	0.0292	1.8744
Junb	1.3184	0.0013	0.0675	2.4939	***Cnbd2***	0.8981	0.0016	0.0755	1.8637
Cytip	1.2802	0.0003	0.0274	2.4288	***Btg3***	0.8914	0.0013	0.0687	1.8549
Pdk4	1.2680	0.0014	0.0697	2.4083	***Col4a1***	0.8877	0.0000	0.0000	1.8502
Agap2	1.2558	0.0000	0.0004	2.3880	***Cyb561***	0.8859	0.0001	0.0160	1.8480
Itgam	1.2532	0.0022	0.0921	2.3837	***Cd300a***	0.8819	0.0004	0.0362	1.8428
Plk3	1.2492	0.0022	0.0914	2.3771	***Cep290***	0.8794	0.0009	0.0549	1.8396
Mt1	1.2491	0.0010	0.0613	2.3770	***C6***	0.8768	0.0000	0.0017	1.8363
Arid5a	1.2415	0.0008	0.0526	2.3644	***Slc6a8***	0.8705	0.0001	0.0120	1.8282
Ccna2	1.2153	0.0008	0.0541	2.3218	***Kctd17***	0.8646	0.0000	0.0069	1.8209
Nnmt	1.1767	0.0003	0.0276	2.2606	***Retreg1***	0.8595	0.0000	0.0052	1.8145
Ercc6l2	1.1738	0.0001	0.0169	2.2560	***Egfr***	0.8583	0.0015	0.0755	1.8129
Tlr13	1.1692	0.0001	0.0194	2.2488	***Cntnap1***	0.8474	0.0008	0.0536	1.7993
Slc3a1	1.1687	0.0014	0.0700	2.2481	***Smpd3***	0.8388	0.0018	0.0812	1.7885
Cd163	1.1658	0.0007	0.0511	2.2436	***Mpzl1***	0.8324	0.0009	0.0552	1.7807
Ccl6	1.1555	0.0000	0.0010	2.2276	***Stat3***	0.8315	0.0000	0.0015	1.7796
Ikzf4	1.1553	0.0008	0.0518	2.2274	***Arl13b***	0.8045	0.0000	0.0061	1.7465
Slc41a2	1.1536	0.0006	0.0425	2.2247	***Zbtb21***	0.8032	0.0000	0.0082	1.7450
Cldn14	1.1474	0.0007	0.0481	2.2151	***Sla***	0.7981	0.0025	0.0984	1.7388
Dock11	1.1307	0.0011	0.0616	2.1897	***Serpina3c***	0.7889	0.0002	0.0242	1.7278
Kif24	1.0983	0.0022	0.0914	2.1410	***Nin***	0.7886	0.0006	0.0427	1.7274
Map3k6	1.0977	0.0011	0.0638	2.1401	***Zfp281***	0.7871	0.0000	0.0084	1.7257
P2ry13	1.0920	0.0018	0.0812	2.1316	***Fbn1***	0.7854	0.0005	0.0405	1.7235
Fndc3b	1.0910	0.0000	0.0000	2.1303	***Golm1***	0.7811	0.0022	0.0914	1.7184
Grem2	1.0706	0.0003	0.0274	2.1003	***Tubb2a***	0.7801	0.0001	0.0177	1.7173
Emilin2	1.0573	0.0010	0.0612	2.0810	***Nlrp12***	0.7779	0.0003	0.0292	1.7146
Fosl2	1.0512	0.0000	0.0104	2.0723	***Atg16l2***	0.7736	0.0000	0.0104	1.7096
Cd207	1.0455	0.0017	0.0788	2.0641	***Tanc2***	0.7733	0.0002	0.0266	1.7092
Csf2rb2	1.0417	0.0013	0.0673	2.0587	***Lrfn3***	0.7710	0.0003	0.0297	1.7065
Klf6	1.0321	0.0000	0.0009	2.0450	***Fbf1***	0.7709	0.0011	0.0629	1.7064
Grhl1	1.0308	0.0015	0.0744	2.0431	***Ccnl1***	0.7672	0.0007	0.0488	1.7020
Slc41a3	1.0284	0.0000	0.0084	2.0397	***Entpd1***	0.7659	0.0005	0.0376	1.7004

Gene	logFC	PValue	FDR	FC	Gene	logFC	PValue	FDR	FC
Nedd9	0.7576	0.0000	0.0063	1.6907	***Olfr1033***	0.6555	0.0000	0.0052	1.5751
Lrg1	0.7554	0.0026	0.0995	1.6880	***Fam124a***	0.6512	0.0004	0.0345	1.5704
Gjc3	0.7536	0.0025	0.0989	1.6860	***Gfra1***	0.6490	0.0001	0.0144	1.5681
Rab30	0.7530	0.0000	0.0017	1.6853	***Ahcyl2***	0.6475	0.0000	0.0052	1.5664
Pla2g7	0.7514	0.0014	0.0711	1.6834	***Acsl4***	0.6449	0.0000	0.0073	1.5636
Cd93	0.7480	0.0007	0.0497	1.6794	***Garem1***	0.6371	0.0000	0.0002	1.5552
Slc16a6	0.7475	0.0000	0.0075	1.6788	***Elk4***	0.6357	0.0000	0.0007	1.5537
Rel	0.7463	0.0000	0.0070	1.6775	***Hspb8***	0.6295	0.0003	0.0309	1.5470
Tsc22d2	0.7445	0.0006	0.0469	1.6754	***Synpo***	0.6287	0.0018	0.0812	1.5461
C8b	0.7404	0.0026	0.0996	1.6707	***Desi2***	0.6281	0.0000	0.0061	1.5455
Anxa1	0.7375	0.0019	0.0833	1.6673	***Snx10***	0.6275	0.0004	0.0332	1.5449
B4galt6	0.7314	0.0006	0.0424	1.6603	***Dst***	0.6269	0.0000	0.0061	1.5442
B4galt4	0.7298	0.0024	0.0976	1.6584	***Ano6***	0.6224	0.0016	0.0779	1.5394
Mbnl2	0.7262	0.0001	0.0165	1.6542	***Ttpal***	0.6167	0.0003	0.0296	1.5334
Olfr344	0.7196	0.0003	0.0296	1.6467	***Mlxip***	0.6157	0.0010	0.0613	1.5323
Rell1	0.7135	0.0023	0.0950	1.6398	***Apobec1***	0.6135	0.0001	0.0136	1.5299
Lad1	0.7107	0.0006	0.0424	1.6365	***Mical2***	0.6123	0.0000	0.0010	1.5287
Bcl3	0.7060	0.0012	0.0646	1.6313	***Flna***	0.6119	0.0024	0.0960	1.5283
Skil	0.7008	0.0000	0.0022	1.6254	***Col4a2***	0.6104	0.0000	0.0000	1.5267
Rgs2	0.6980	0.0010	0.0595	1.6223	***Onecut2***	0.6089	0.0000	0.0015	1.5251
Slc25a22	0.6870	0.0004	0.0351	1.6100	***Stk17b***	0.6059	0.0027	0.0998	1.5219
Apcs	0.6857	0.0002	0.0204	1.6085	***Gldc***	0.6057	0.0000	0.0009	1.5217
Neu3	0.6852	0.0013	0.0673	1.6080	***Fabp5***	0.6053	0.0018	0.0812	1.5213
Slc41a1	0.6771	0.0023	0.0950	1.5990	***Nedd4l***	0.6049	0.0005	0.0373	1.5208
Myh10	0.6760	0.0000	0.0017	1.5977	***Cyp4a12b***	0.6000	0.0001	0.0119	1.5157
Insig2	0.6698	0.0000	0.0052	1.5909	***Ptp4a1***	0.5998	0.0009	0.0552	1.5155
Dgkh	0.6682	0.0000	0.0052	1.5891	***Sh3pxd2a***	0.5915	0.0012	0.0646	1.5068
Col12a1	0.6619	0.0023	0.0938	1.5822	***Msr1***	0.5900	0.0017	0.0805	1.5053
Fam120c	0.6609	0.0003	0.0316	1.5810	***Clpx***	0.5881	0.0015	0.0726	1.5032
Setd7	0.6597	0.0001	0.0127	1.5797	***Tanc1***	0.5866	0.0002	0.0197	1.5017
Eppk1	0.6592	0.0000	0.0039	1.5792	***Wnk1***	0.5864	0.0004	0.0365	1.5015
Fyb2	0.6590	0.0012	0.0667	1.5789					
Tor3a	0.6560	0.0022	0.0921	1.5757					
Cntrl	0.6555	0.0000	0.0005	1.5752					

Gene	logFC	PValue	FDR	FC	Gene	logFC	PValue	FDR	FC
Gm20388	-9.4576	0.0001	0.0193	0.0014	***D130043K22Rik***	-0.8995	0.0012	0.0646	0.5361
Olfr1565	-6.8619	0.0000	0.0024	0.0086	***Purg***	-0.8973	0.0016	0.0777	0.5369
Cphx3	-6.7902	0.0010	0.0613	0.0090	***Izumo4***	-0.8815	0.0007	0.0488	0.5428
BC035947	-6.5240	0.0002	0.0205	0.0109	***Mid1ip1***	-0.8733	0.0025	0.0983	0.5459
Vmn1r53	-4.4005	0.0004	0.0345	0.0474	***Dyrk3***	-0.8726	0.0015	0.0720	0.5461
Noxa1	-3.9605	0.0009	0.0552	0.0642	***Adamts7***	-0.8606	0.0002	0.0203	0.5507
Lrrc71	-3.2579	0.0009	0.0570	0.1045	***Sult1c2***	-0.8566	0.0002	0.0242	0.5523
4930513O06Rik	-2.5169	0.0001	0.0119	0.1747	***Ces1d***	-0.8507	0.0001	0.0120	0.5545
Hao2	-1.7975	0.0022	0.0914	0.2877	***Lrit2***	-0.8387	0.0000	0.0017	0.5591
Ms4a12	-1.7914	0.0014	0.0711	0.2889	***Cyp26a1***	-0.8008	0.0000	0.0020	0.5740
Cnmd	-1.6905	0.0000	0.0052	0.3098	***Kcnk5***	-0.7871	0.0018	0.0812	0.5795
Hamp2	-1.6859	0.0000	0.0002	0.3108	***Impg2***	-0.7713	0.0000	0.0102	0.5859
Dsg1c	-1.6295	0.0000	0.0000	0.3232	***Gas1***	-0.7483	0.0001	0.0109	0.5953
Mycn	-1.5707	0.0006	0.0460	0.3366	***Nr1i3***	-0.7267	0.0000	0.0104	0.6043
Lrrc15	-1.5212	0.0000	0.0009	0.3484	***Gstt2***	-0.7180	0.0010	0.0610	0.6079
Acot3	-1.3758	0.0004	0.0367	0.3853	***1810058I24Rik***	-0.7146	0.0001	0.0144	0.6094
Nat8f7	-1.3416	0.0002	0.0245	0.3946	***Dbp***	-0.6854	0.0005	0.0408	0.6218
Nr0b2	-1.1858	0.0015	0.0735	0.4396	***Hpgd***	-0.6723	0.0000	0.0015	0.6275
Slc6a16	-1.1068	0.0007	0.0474	0.4643	***Akr1c19***	-0.6702	0.0000	0.0001	0.6284
Serpina6	-1.0795	0.0000	0.0000	0.4732	***4930579F01Rik***	-0.6599	0.0002	0.0250	0.6329
Inmt	-1.0789	0.0000	0.0003	0.4734	***Syt3***	-0.6535	0.0017	0.0800	0.6357
Serpinb1a	-1.0587	0.0013	0.0684	0.4800	***Fdft1***	-0.6498	0.0000	0.0061	0.6374
Tmem138	-1.0231	0.0022	0.0934	0.4920	***Tk1***	-0.6428	0.0001	0.0160	0.6404
Car1	-1.0204	0.0001	0.0136	0.4930	***Aifm3***	-0.6228	0.0005	0.0410	0.6494
Cyp46a1	-0.9969	0.0003	0.0292	0.5011	***Pnpla1***	-0.6181	0.0024	0.0968	0.6515
D930048N14Rik	-0.9904	0.0008	0.0517	0.5033	***Zfp606***	-0.6044	0.0021	0.0896	0.6578
Dclk3	-0.9711	0.0001	0.0166	0.5101	***Ak2***	-0.6023	0.0000	0.0016	0.6587
Cyp2a5	-0.9581	0.0000	0.0000	0.5147	***Car3***	-0.5993	0.0004	0.0365	0.6601
Slco1a4	-0.9333	0.0000	0.0015	0.5237	***Zfp467***	-0.5983	0.0005	0.0420	0.6605
Hsd17b6	-0.9171	0.0002	0.0197	0.5296	***Slc2a5***	-0.5953	0.0016	0.0783	0.6619
Map2k6	-0.9136	0.0006	0.0444	0.5308	***Haus4***	-0.5912	0.0000	0.0073	0.6638

FC: Fold Change; FDR: False Discovery rate

Supplemental Table 3. PANDORA-seq detects significantly changed sperm rsRNAs and tsRNAs induced by DCHP exposure

Sequence	Length	Annotation	fold_change	q_value (%)
TTCGTACATCTAGGAGCTATAGAACTAGTACCGCAAGGGAAAG	43	16S-rRNA	2.5011	7.7906
TTCTTAGTTGGTGGAG	16	18S-rRNA	2.0982	0.0000
TCTTAGTTGGTGGAG	15	18S-rRNA	2.1913	0.0000
TTTGGTGACTCTAGATAACCTCGGGCCGATCGCACGCCCC	40	18S-rRNA	2.0205	5.8429
TTAGTTGGTGGAGCGAT	17	18S-rRNA	2.3755	5.8429
GGTTAATTCCGATAACGAACGAGACTCTGGCATGCTAACTAGT	43	18S-rRNA	2.5590	5.8429
TTAGTTGGTGGAGCGATTTGTCTGGTTAATTCCGATAACGAACG	44	18S-rRNA	2.1973	7.7906
GTCTGGTTAATTCCGATAACGAACGAGACTCTGGCATGCTAAC	43	18S-rRNA	2.1106	7.7906
TGGTTAATTCCGATAACGAACGAGACTCTGGCATGCTAACTAG	43	18S-rRNA	2.5648	7.7906
TCTTAGTTGGTGGAGCGATTTGTCTGGTTAATTCCGATAACGAA	44	18S-rRNA	2.7403	7.7906
AGACGGACCAGAGCGAAAGCATTTGCCAAGAATGTTTTCATTAAT	45	18S-rRNA	2.0833	8.1270
TCTTAGTTGGTGGAGCGAT	19	18S-rRNA	2.1012	8.1270
TTAGTTGGTGGAGCGATTTGTCTGGTTAATTCCGATAACGAA	42	18S-rRNA	2.8972	8.1270
CTTAGTTGGTGGAGCGAT	18	18S-rRNA	2.3625	8.1270
TGGTTAATTCCGATAACGAACGAGACTCTGGCATGCTAACTAGT	44	18S-rRNA	2.1005	8.1270
GGGGCGGGGGGCCCGGA	17	28S-rRNA	11.1189	0.0000
GTGTGGAACCTGGCGCTAAACCATTCGTAGACGACCTGCTT	41	28S-rRNA	2.0208	0.0000
ATTCAAACGAGAACTTTGAAGGCCGAAGTGGAGAAGGGTTCCA	43	28S-rRNA	2.3532	0.0000
TGGCGACCCGCTGAA	15	28S-rRNA	2.2478	5.8429
TGGCGTGGAGCCGGGC	16	28S-rRNA	9.6442	5.8429
TCCTCCCGACCCCTC	15	28S-rRNA	3.0462	7.7906
GCAAATCGGTCGTCCGACCTGGGTATAGGGGCGAAAGAC	39	28S-rRNA	2.0118	7.7906
CTCGCTGCGATCTATTGAAAGTCAGCCCTCGACACAAGGGT	41	28S-rRNA	2.0725	7.7906
GATTTCTGCCCAGTGCTCTGAATGTCAAAGTGAAGAAATTCAAT	44	28S-rRNA	2.9102	7.7906
AATTAGTGACGCGCATGAATGGATGAACGAGATTCCCAC	39	28S-rRNA	2.0365	7.7906
ACGTTGAACGCACGTTCGTGTGGAACCTGGCGCTAAACCATTCG	44	28S-rRNA	2.3707	7.7906
AGACGACCTGCTTCTGGGTCGGGGTTTCGTACGTAGCAGAGCAG	44	28S-rRNA	2.3127	7.7906
GTGTGGAACCTGGCGCTAGACCAT	24	28S-rRNA	3.4860	7.7906
GTGTGGAACCTGGCGCTAAACCAT	24	28S-rRNA	2.4817	7.7906
GAGCCAATGGGGCGAAGCTACCATCTGTGGGATTATGACTGAA	43	28S-rRNA	2.3690	7.7906
TGAAACACGGACCAAGGAGTCTAACGCGTGCGCGAGTCAGGGGCT	45	28S-rRNA	2.8513	7.7906
ATTCAAACGAGAACTTTG	18	28S-rRNA	2.5514	7.7906

GTTGAACGCACGTTCGTGTGGAACCTGGCGCTAAACCATTCG	42	28S-rRNA	2.2064	7.7906
GTGTGGAACCTGGCGCTAAACCATTCGTAGACGACCTGCT	40	28S-rRNA	2.0488	7.7906
GGGGGGACCGTCGGT	15	28S-rRNA	4.0398	7.7906
ACGTTCGTGTGGAAC	15	28S-rRNA	2.6031	8.1270
GAAACACGGACCAAGGAGTCTAACGCGTGCGCGAGTCAGGGGCT	44	28S-rRNA	2.7348	8.1270
TTGACTCTAGTCTGGCACGGTGAAGAGACATGAGAGGTGTAGAA	44	28S-rRNA	3.0585	8.1270
CGCGACCTCAGATCAGA	17	28S-rRNA	2.2721	8.1270
TGGAGCACGAGCGTACGCGTTAGGACCCGAAAGATGGTGAA	41	28S-rRNA	2.6925	8.1270
TAGACGACCTGCTTCTGGGTCGGGGTTTCGTACGTAGCAGAGCAG	45	28S-rRNA	2.0220	8.1270
AGGACGGTGGCCATGGAAGTCGGAATCCGCTAAGGAGTGTGTAA	44	28S-rRNA	2.6189	8.1270
ACGTTCGTGTGGAACCTGGCGCTAAACCAT	30	28S-rRNA	2.1311	8.1270
CGCGACCTCAGATCAGACGTGGCGACCCGCTGAATTTAAGCATA	44	28S-rRNA	2.1629	8.1270
GGAGCACGAGCGTACGCGTTAGGACCCGAAAGATGGTGAA	40	28S-rRNA	2.2785	8.1270
GAGCCAATGGGGCGAAGCTACCATCTGTGGGATTATGACTGAACG	45	28S-rRNA	2.4328	8.1270
ATCCTTCGATGTCGGCTCTTCCTATCATTGTGAAGCAGAATTCAC	45	28S-rRNA	2.7807	8.1270
AGCCGACTTAGAACTGGTGCGGACCAGGGGAATCCGACTGTC	42	28S-rRNA	2.0335	8.1270
AACCGACTTAGAACTGGTGCGGACCAGGGGAATCCGACTGTT	42	28S-rRNA	2.2615	8.1270
CGCGACCTCAGATCAG	16	28S-rRNA	2.2767	8.1270
AAACGAGAACTTTGAA	16	28S-rRNA	3.5657	8.1270
ATTAGTGACGCGCATGAATGGATGAACGAGATTCCCAC	38	28S-rRNA	2.2235	8.1270
AGCCGACTCAGAACTGGTGCGGACCAGGGGAATCCGACTGTT	42	28S-rRNA	2.1549	8.1270
TGGGCCACTTTTGGTAAGCAGAACTGGCGCTGCGGGATGAA	41	28S-rRNA	2.6329	8.1270
AGGACACATTGATCATCGACACTTCGAACGCACTTGCGGCCCCG	44	5.8S-rRNA	2.0234	8.1270
AAAAACATTAGACTGTGAATCTGACAACAGGAAATAAACCTCCTT	45	mature-mt_tRNA-His-GTG	4.6325	0.0000
AAAACATTAGACTGTGAATCTGACAACAGGAAATAAACCTCCTT	44	mature-mt_tRNA-His-GTG	4.9751	0.0000
AAAACATTAGACTGTGAATCTGACAACAGGAAATAAACCTCCT	43	mature-mt_tRNA-His-GTG	4.8024	5.8429
AAAACATTAGACTGTGAATCTGACAACAGGAAATAAACCTCCTTA	45	mature-mt_tRNA-His-GTG	4.1089	5.8429
AAACATTAGACTGTGAATCTGACAACAGGAAATAAACCTCCTTA	44	mature-mt_tRNA-His-GTG	5.1753	5.8429
AAAAACATTAGACTGTGAATCTGACAACAGGAAATAAACCTCCT	44	mature-mt_tRNA-His-GTG	5.2256	7.7906
AAACATTAGACTGTGAATCTGACAACAGGAAATAAACCTCCT	42	mature-mt_tRNA-His-GTG	4.7049	7.7906
AAACATTAGACTGTGAATCTGACAACAGGAAATAAACCTCCTT	43	mature-mt_tRNA-His-GTG	5.3681	7.7906
TAGACTGTGAATCTGACAACAGGAAATAAACCTCCTTATTCACCC	45	mature-mt_tRNA-His-GTG	4.1905	7.7906
CAAAAAACATTAGACTGTGAATCTGACAACAGGAAATAAACCTCC	45	mature-mt_tRNA-His-GTG	5.0375	8.1270
AGTTTACAAAAAACATTAGACTGTGAATCTGACAACAGG	39	mature-mt_tRNA-His-GTG	4.9983	8.1270
AAGGCATTGGCCTCCTAAGCCAGGGATTGTGGGTT	35	mature-tRNA-Arg-CCT	2.3384	7.7906

AAGGCACTGGCCTCCTAAGCCAGGGATTGTGGGTT	35	mature-tRNA-Arg-CCT	2.2728	7.7906
ATTGGCCTCCTAAGCCAGGGATTGTGGGTT	30	mature-tRNA-Arg-CCT	3.3051	8.1270
GCATTGGTGGTTCAGTGGTAGAAT	24	mature-tRNA-Gly-GCC_5_end;mature-tRNA-Gly-CCC_5_end	2.8288	8.1270
GGTAGCGTGGCCGAG	15	mature-tRNA-Leu-AAG_5_end;mature-tRNA-Leu-TAG_5_end	5.5337	0.0000
GGTAGCGTGGCTGAG	15	mature-tRNA-Leu-AAG_5_end;mature-tRNA-Leu-TAG_5_end	3.0582	8.1270

Supplemental Figure 1. Uncropped Western blot images

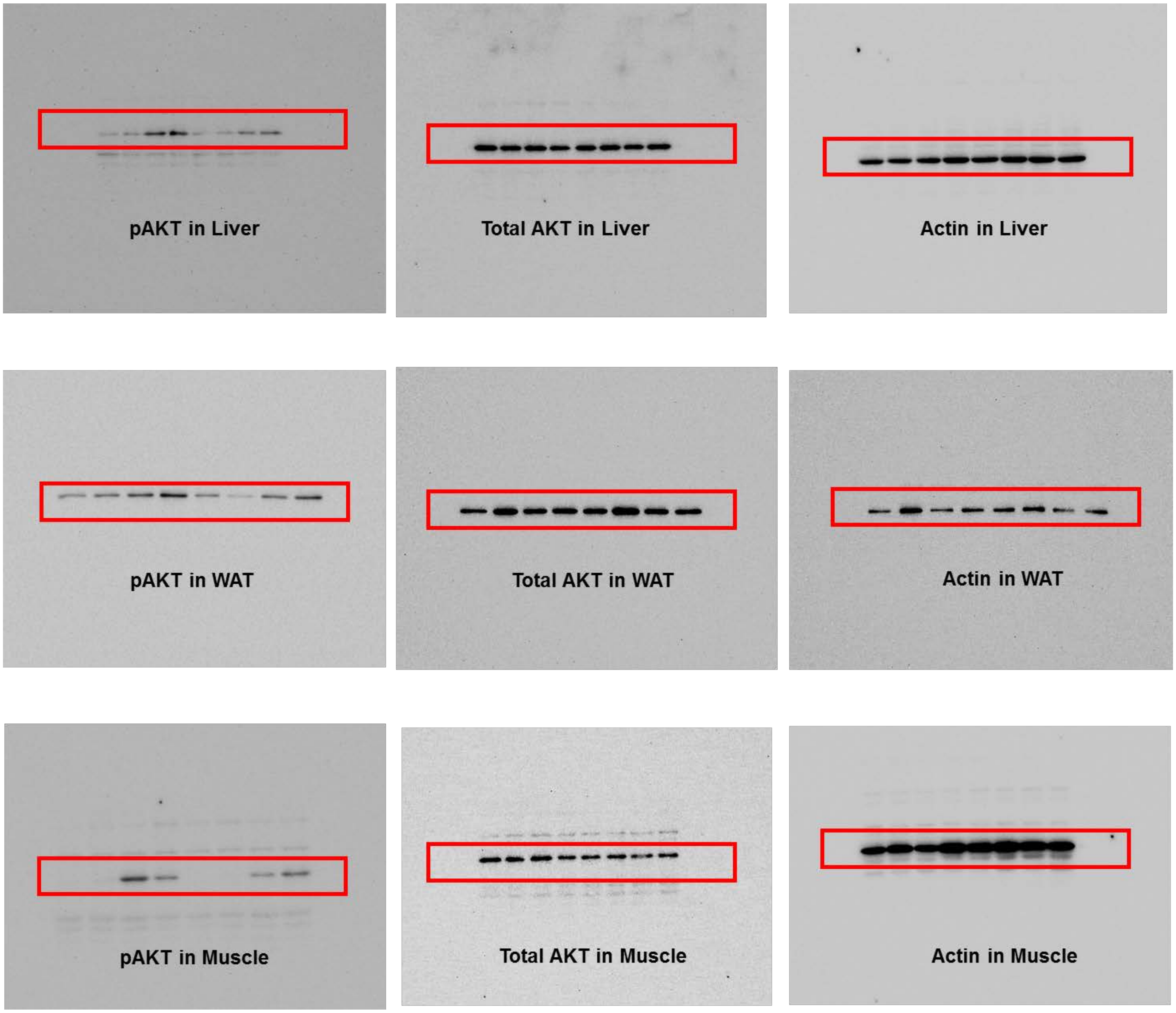

Supplement: Supplementary Material [file NIHMS1890150-supplement-Supplementary_Material.pdf]
